# 中国成人急性淋巴细胞白血病诊断与治疗指南（2024年版）

**DOI:** 10.3760/cma.j.cn121090-20240319-00102

**Published:** 2024-05

**Authors:** 中国抗癌协会血液肿瘤专业委员会 中华医学会血液学分会白血病淋巴瘤学组

**Keywords:** 白血病，淋巴细胞，急性, 成人, 诊断, 治疗, 疗效监测, Leukemia, lymphoblastic, acute, Adult, Diagnosis, Treatment, Efficacy monitoring

## Abstract

成人急性淋巴细胞白血病（ALL）是成人最常见的急性白血病之一，目前国际上有比较统一的诊断标准和不同研究组报道的系统治疗方案。我国自2013年至今发表了三版中国成人ALL诊断与治疗的专家共识/指南，对该病种诊断治疗水平提升有重要意义。2022年WHO造血与淋巴组织肿瘤分类对ALL（前体淋巴细胞肿瘤——淋巴母细胞白血病/淋巴瘤）的分类进行了更新，提出了一些新概念；近年来免疫治疗快速发展，在一定程度上提高了成人ALL的疗效，且抗体、嵌合抗原受体T细胞（CAR-T细胞）产品陆续在国内上市，临床实践日益丰富。为促进成人ALL临床诊疗的规范，本指南参考国内外最新指南和文献，荟萃国内外最新研究成果，为成人ALL的诊断、治疗、疗效监测提供参考意见。

## Abstract (en)

Adult acute lymphoblastic leukemia（ALL）is one of the most common acute leukemia in adults. There are relatively unified diagnostic criteria and systematic treatment regimens reported by different research groups in the world. Since 2013, three versions of expert consensus/guidelines on the diagnosis and treatment of adult ALL in China have been published, which are of great significance for improving the level of diagnosis and treatment of this disease. In 2022, the classification of ALL（precursor lymphocyte neoplasms-lymphoblastic leukemias/lymphomas）had been updated in the WHO classification of haematolymphoid tumors, and some new concepts had been proposed. In recent years, the rapid development of immunotherapy has improved the efficacy of adult ALL, and commercial antibodies and CAR-T cell products have been available in China, the clinical practice is increasing. In order to promote the standardization of clinical diagnosis and treatment of adult ALL, by referring to the latest guidelines and literatures all over the world, this guideline may contribute to the better understanding of diagnosis, treatment and efficacy monitoring for adult ALL.

成人急性淋巴细胞白血病（ALL）是成人最常见的急性白血病之一，占成人急性白血病的20％～30％，目前国际上有比较统一的诊断标准和不同研究组报道的系统治疗方案，完全缓解（CR）率可达70％～90％，3～5年无病生存（DFS）率达30％～60％。美国国家癌症综合网（NCCN）于2012年首次公布了成人ALL的诊断治疗指南，并逐年更新；我国分别于2013年、2016年及2021年发表了三版中国成人ALL诊断与治疗的专家共识/指南[1]，得到了国内同行的认可。2022年WHO造血与淋巴组织肿瘤分类对ALL（前体淋巴细胞肿瘤——淋巴母细胞白血病/淋巴瘤）的分类进行了更新，提出了一些新概念；近年来免疫治疗快速发展，在一定程度上提高了成人ALL的疗效，且抗体、嵌合抗原受体T细胞（CAR-T细胞）产品陆续在国内上市，临床实践日益丰富[2]–[3]。基于此，中国成人ALL诊治指南进行再次更新。

Burkitt淋巴瘤/白血病（BL）临床上常以骨髓血液受累起病，细胞增殖速度快、侵袭性高，表现为急性白血病的特点；治疗方案多优先采用联合CD20单克隆抗体（利妥昔单抗）的短疗程、短间隔治疗。但鉴于WHO造血及淋巴组织肿瘤分类已将其归入成熟B细胞肿瘤，中国2021年ALL指南没有再纳入这一疾病（可以参见Burkitt淋巴瘤/白血病相关指南）。

## 第一部分 ALL的诊断分型

一、概述

ALL诊断应采用MICM（细胞形态学、免疫学、细胞遗传学和分子遗传学）诊断模式[4]，诊断分型采用WHO 2022（第5版）标准。最基本的检查应包括细胞形态学、免疫表型，以保证ALL与急性髓系白血病（AML）等的鉴别，初诊时应行骨髓穿刺+骨髓活检；骨髓中原始/幼稚淋巴细胞比例≥20％才可以诊断ALL（少数患者因发热、使用糖皮质激素可导致原始细胞比例不足20％，需要结合病史和其他检查鉴别诊断）。骨髓干抽者可考虑采用外周血和骨髓活检（应进行免疫组化检查）进行必要的检查。为准确判断肿瘤负荷，可酌情考虑相关的影像学检查（B超、CT等）。

免疫分型应采用多参数流式细胞术，最低诊断分型可以参考欧洲白血病免疫分型协作组（EGIL）标准（表1）。同时应除外系列不清的急性白血病（尤其是混合表型急性白血病），混合表型急性白血病的系列确定建议参照WHO 2008/2016造血及淋巴组织肿瘤分类的标准（表2），可以同时参考EGIL标准（表3），早期T前体淋巴母细胞白血病（ETP-ALL）免疫表型诊断积分可参考表4。

**表1:** 急性淋巴细胞白血病（ALL）的免疫学分型（EGIL，1995）

亚型	免疫学标准
B系ALL^a^	CD19、CD79a、CD22至少两个阳性
早期前B-ALL（B-Ⅰ）	无其他B细胞分化抗原表达
普通型ALL（B-Ⅱ）	CD10^+^
前B-ALL（B-Ⅲ）	胞质IgM^+^
成熟B-ALL（B-Ⅳ）	胞质或膜κ或λ^+^
T系ALL^b^	胞质/膜CD3^+^
早期前T-ALL（T-Ⅰ）	CD7^+^
前T-ALL（T-Ⅱ）	CD2^+^和（或）CD5^+^和（或）CD8^+^
皮质T-ALL（T-Ⅲ）	CD1a^+^
成熟T-ALL（T-Ⅳ）	膜CD3^+^，CD1a^−^
α/β^+^ T-ALL（A组）^c^	抗TCRα/β^+^
γ/δ^+^ T-ALL（B组）^c^	抗TCRγ/δ^+^
伴髓系抗原表达的ALL（My^+^ALL）	表达1或2个髓系标志，但又不满足混合表型急性白血病的诊断标准

**注** ^a^绝大多数B-ALL患者TdT和HLA-DR阳性（B-Ⅳ除外，TdT多为阴性）；^b^绝大多数T-ALL患者TdT阳性，HLA-DR、CD34为阴性（但不作为诊断分类必需）；^c^T-ALL中根据膜表面T细胞受体（TCR）的表达情况进行的分组

**表2:** WHO 2022分类标准对系列诊断的要求

系列	诊断要求
髓系	髓过氧化物酶阳性（强度部分超过成熟中性粒细胞水平的50%）
	或
	单核细胞分化（NSE、CD11c、CD14、CD64、溶菌酶，至少2项表达）
T细胞系	CD3阳性（胞质或膜），流式细胞术检测显示强度部分超过成熟T细胞水平的50%或用非ζ链试剂行免疫细胞化学检测显示阳性
B细胞系	CD19强表达，CD79a、CD22、CD10至少一种强阳性
	或
	CD19弱表达，CD79a、CD22、CD10至少两种强阳性

**表3:** EGIL急性混合型白血病的诊断积分系统（EGIL，1998）

积分	B细胞系	T细胞系	髓系
2	CD79a	Cy/mCD3	MPO
	CyIgM	抗TCRα/β	
	CyCD22	抗TCRγ/δ	
1	CD19	CD2	CD117
	CD20	CD5	CD13
	CD10	CD8	CD33
		CD10	CDw65
0.5	TdT	TdT	CD14
	CD24	CD7	CD15
		CD1a	CD64

**注** 每一系列2分才可以诊断

**表4:** 早期T前体淋巴母细胞白血病（ETP-ALL）免疫表型诊断积分（在CD7阳性、cCD3阳性的基础上，该积分≥8分诊断ETP-ALL）[10]

免疫表型	表达	不表达
CD1a	−2	2
sCD3	−2	
CD5	−2	2
CD8		2
CD10		1
CD13	1	
CD33	1	
CD34	1	
CD117	1	
TdT		1
MPO	−3	

**注** 表达定义为至少20％白血病细胞染色阳性（CD5、MPO除外），CD5表达定义为≥75％白血病细胞染色阳性，MPO表达定义为≥3％白血病细胞染色阳性（细胞化学和/或流式细胞术检测）

为保证诊断分型的准确性、预后判断合理可靠，应常规进行遗传学检查，包括染色体核型分析及必要的荧光原位杂交（FISH）检查，如KMT2A、CRLF2、JAK2等基因重排和TP53基因缺失。开展相关的分子学检测（融合基因筛查、BCR::ABL1样ALL的筛查，有条件的单位可考虑开展转录组测序），以满足ALL精准分型；建议开展二代测序技术（NGS）检测基因突变和基因拷贝数变异（如IKZF1和CDKN2A/B缺失等），为患者诊断分型、预后判断、靶向治疗提供依据[5]–[10]。预后分组可参考NCCN 2023临床危险度分组和细胞遗传学预后分组标准（表5、表6）。

**表5:** 成人急性淋巴细胞白血病（ALL）临床预后危险分层

因素	高危	
	B-ALL	T-ALL
年龄	>35岁	>35岁
WBC	>30×10^9^/L	>100×10^9^/L
免疫表型	N/A	ETP-ALL
细胞遗传学/分子生物学危险度	见表6	RAS/PTEN突变和（或）NOTCH1/FBXW7野生型
CR后MRD	阳性/≥10^−4^	阳性/≥10^−4^

**注** CR：完全缓解；MRD：可测量残留病；ETP-ALL：早期T前体淋巴母细胞白血病；N/A：不适用

**表6:** 成人急性B淋巴细胞白血病的细胞遗传学和分子生物学预后分组

危险度分组	细胞遗传学和分子学改变
标危组	高超二倍体(51～65条染色体；4、10、17三体预后最好)
	t(12;21)(p13;q22)/ETV6::RUNX1
	t(1;19)(q23;p13.3)/TCF3::PBX1
	DUX4重排
	PAX5 P80R
	t(9;22)(q34;q11.2)/BCR::ABL1^a^不伴IKZF1 plus^b^且无慢性髓性白血病(CML)病史
高危组	低二倍体(<44条染色体)
	TP53突变
	KMT2A重排：t(4;11)或其他
	IgH重排
	HLF重排
	ZNF384重排
	MEF2D重排
	MYC重排
	BCR::ABL1样（Ph样）ALL
	·JAK-STAT（CRLF2、EPOR、JAK1/2/3、TYK2重排；SH2B3、IL7R、JAK1/2/3突变）
	·ABL同源激酶重排（如ABL1、ABL2、PDGFRA、PDGFRB、FGFR1）
	·其他（NTRK3、FLT3、LYN、PTK2B重排）
	PAX5改变
	t(9;22)(q34;q11.2)：BCR::ABL1伴IKZF1 plus和（或）CML病史
	21号染色体内部扩增(iAMP21)
	IKZF1改变
	复杂染色体异常(携带5种及5种以上染色体异常，不伴有上述常见染色体易位/融合基因、分子异常和倍体异常)

**注** ^a^有条件的医疗机构可通过FISH检测外周血中性粒细胞是否存在BCR::ABL1以鉴别初诊慢性髓性白血病（CML）急变和初诊Ph^+^-ALL。^b^IKZF1 plus指IKZF1缺失伴CDKN2A、CDKN2B、PAX5缺失，或无ERG缺失时存在PAR1缺失

ALL诊断确立后，应根据患者的具体分型、预后分组，采用规范化的分层治疗策略，以取得最佳治疗效果。

二、WHO 2022（第5版）关于前体淋巴细胞肿瘤分类[3]

1. B淋巴母细胞白血病/淋巴瘤（B-ALL/LBL）：

（1）B淋巴母细胞白血病/淋巴瘤，非特指型（NOS）

（2）伴重现性遗传学异常的B淋巴母细胞白血病/淋巴瘤：

•伴BCR::ABL1融合基因的B淋巴母细胞白血病/淋巴瘤

•伴KMT2A重排的B淋巴母细胞白血病/淋巴瘤

•伴ETV6::RUNX1融合基因的B淋巴母细胞白血病/淋巴瘤

•伴高超二倍体的B淋巴母细胞白血病/淋巴瘤

•伴亚二倍体的B淋巴母细胞白血病/淋巴瘤

•伴IGH::IL3融合基因的B淋巴母细胞白血病/淋巴瘤

•伴TCF3::PBX1融合基因的B淋巴母细胞白血病/淋巴瘤

•伴TCF3::HLF融合基因的B淋巴母细胞白血病/淋巴瘤[5]–[6]

•伴iAMP21的B淋巴母细胞白血病/淋巴瘤

•BCR::ABL1样B淋巴母细胞白血病/淋巴瘤

•ETV6::RUNX1样B淋巴母细胞白血病/淋巴瘤[7]

（3）建议分类：伴其他确定基因异常的B淋巴母细胞白血病/淋巴瘤

2. T淋巴母细胞白血病/淋巴瘤（T-ALL/LBL）：根据抗原表达划分为不同的阶段：pro-T、pre-T、皮质-T、髓质-T。

（1）T淋巴母细胞白血病/淋巴瘤，非特指型（NOS）

（2）早期T前体淋巴母细胞白血病/淋巴瘤（Early T-cell precursor lymphoblastic leukemia/lymphoma，ETP-ALL/LBL）。

注：临床一般采用“急性淋巴细胞白血病（ALL）”替代“淋巴母细胞白血病”。KMT2A＝MLL，ETV6::RUNX1＝TEL::AML1，TCF3::PBX1＝E2A::PBX1。

三、几种特殊类型ALL的特点

1. BCR::ABL1样ALL/LBL（BCR::ABL1-like ALL/LBL）[9]：

（1）与BCR::ABL1阳性（Ph阳性）ALL患者具有相似的基因表达谱。

（2）共同特征是涉及其他酪氨酸激酶基因的易位、CRLF2易位。还包括EPOR（EPO受体）截短重排、激活等少见情况。CRLF2易位患者常与JAK基因突变有关。

（3）涉及酪氨酸激酶基因的易位可以累及ABL1（伙伴基因并非BCR）、ABL2、PDGFRA、PDGFRB、NTRK3、TYK2、CSF1R、JAK2等，形成多种融合基因。

（4）IKZF1和CDKN2A/B缺失发生率较高。

BCR::ABL1样ALL的筛查流程建议见图1。

**图1:**
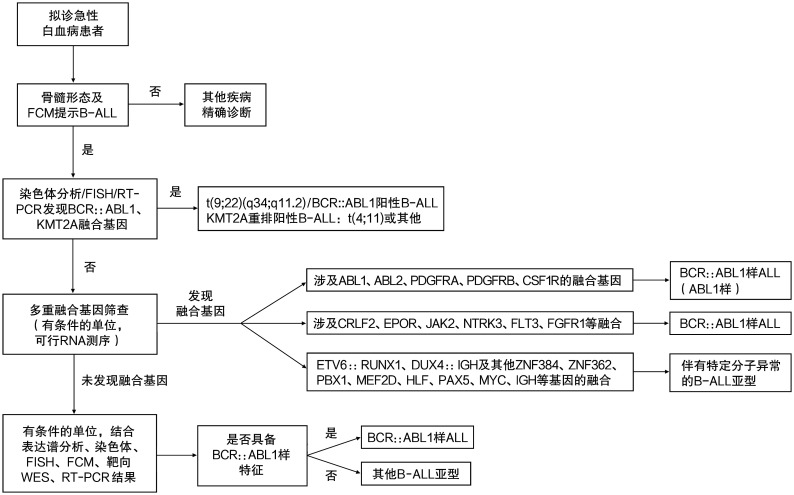
BCR::ABL1样急性淋巴细胞白血病（ALL）的筛查流程图 **注** FCM：流式细胞术；FISH：荧光原位杂交；WES：全外显子测序

2. ETP-ALL/LBL：

（1）CD7阳性，CD1a和CD8阴性。

（2）cCD3阳性（膜CD3阳性罕见），CD2和（或）CD4可以阳性。CD5一般阴性，或阳性率<75％。

（3）髓系/干细胞抗原CD34、CD117、HLA-DR、CD13、CD33、CD11b或CD65一个或多个阳性；MPO阴性（免疫表型积分见表4）。

（4）常伴有髓系白血病相关基因突变：FLT3、NRAS/KRAS、DNMT3A、IDH1和IDH2等。

（5）T-ALL常见的异常，如NOTCH1、CDKN1/2A突变不常见。

3. ETV6::RUNX1样B淋巴母细胞白血病/淋巴瘤[7]：与ETV6::RUNX1阳性ALL患者具有相似的基因表达谱,但是通过FISH、RT-PCR、RNA-seq等检测均未发现ETV6::RUNX1融合基因，这类患者通常同时存在ETV6（形成其他融合基因或缺失）和IKZF1异常，ETV6异常结合IKZF1异常可以引起与经典ETV6::RUNX1融合基因阳性患者相同的转录异常。

4. 伴其他确定基因异常的B淋巴母细胞白血病/淋巴瘤：包括未来有可能成为新分类但目前证据尚不足的基因异常，如伴DUX4重排、MEF2D重排、ZNF384重排、NUTM1重排、IG::MYC融合基因、PAX5改变（重排、基因内扩增或突变）或PAX5 p.P80R等异常。

## 第二部分 成人ALL的治疗

患者一经确诊应尽快开始治疗，应根据疾病分型、遗传学特征、临床预后因素及患者耐受性采用合适的治疗方案、策略。

ALL的治疗分为诱导治疗（部分病例需要预治疗）、缓解后的巩固强化治疗、维持治疗等几个阶段及髓外白血病［主要是中枢神经系统白血病（CNSL）］的预防和治疗。

一、Ph^−^-ALL的治疗

（一）诱导治疗

1. 治疗原则：年轻成人和青少年患者（<40岁，AYA）：①临床试验；②儿童特点联合化疗方案（优先选择）；③多药联合化疗方案。

成年患者（≥40岁）：①<60岁的患者，可以入组临床试验，或采用多药联合化疗；②≥60岁的患者，可以入组临床试验，或采用多药化疗诱导治疗，或糖皮质激素±长春碱类为基础的姑息治疗。

鼓励开展有科学依据的探索性前瞻性临床试验。

2. 治疗方案：一般以4周方案为基础。年轻成人和非老年ALL至少应予长春新碱（VCR）或长春地辛、蒽环/蒽醌类药物［如柔红霉素（DNR）、去甲氧柔红霉素（IDA）、阿霉素、米托蒽醌等］、糖皮质激素（如泼尼松、地塞米松等）为基础的方案（如VDP、VIP）诱导治疗。CD20表达阳性的患者可联合CD20单克隆抗体（利妥昔单抗）。

推荐采用VDP联合门冬酰胺酶（ASP：大肠杆菌或欧文菌来源，或培门冬酰胺酶）［可再联合环磷酰胺（CTX）］组成的VD（C）LP方案，鼓励开展临床研究。也可以采用Hyper-CVAD方案。

蒽环/蒽醌类药物：可以连续应用（连续2～3 d，第1、3周；或仅第1周用药）；也可以每周用药1次（每周的第1天）。

用药参考剂量：DNR 30～45 mg·m^−2^·d^−1^、IDA 6～10 mg·m^−2^·d^−1^、米托蒽醌6～10 mg·m^−2^·d^−1^。

3. 注意事项：

（1）预治疗：WBC≥30×10^9^/L，或者肝、脾、淋巴结肿大明显；或有发生肿瘤溶解特征（生化、电解质检查等结果）的患者进行预治疗，以防止肿瘤溶解综合征的发生。

预治疗方案：糖皮质激素（如泼尼松或地塞米松等，按泼尼松1 mg·kg^−1^·d^−1^口服或静脉用，连续3～5 d）。可以联合应用CTX（200 mg·m^−2^·d^−1^，静脉滴注，连续3～5 d）。

（2）单次应用CTX剂量较大时（超过1 g）可以予美司钠解救。

（3）诱导治疗第14天复查骨髓，根据骨髓情况（增生程度、原始细胞比例等）、血常规及并发症情况调整第3周的治疗（是否需要继续用DNR和CTX）。

一般于诱导治疗第28（＋7）天评估疗效，包括骨髓形态学和可测量残留病（minimal/measurable residual disease，MRD）水平，未能达CR/血细胞未完全恢复的CR（CRi）的患者进入挽救治疗。

（4）尽早开始腰椎穿刺（腰穿）、鞘内注射（鞘注），预防CNSL（可在血小板计数安全水平、外周血没有原始细胞时进行）。

（5）60岁以上的老年患者根据体能状态评估可以采用长春碱类+糖皮质激素，或长春碱类+糖皮质激素+巯嘌呤（6-MP）+甲氨蝶呤（MTX）组成的POMP低强度治疗方案。也可以应用miniHCVD方案或长春碱类+蒽环类药+CTX+ASP+糖皮质激素等药物的多药化疗方案（中高强度治疗），酌情调整药物剂量。

体能状态较差、伴严重感染（不适合常规治疗）的非老年患者也可以采用低强度治疗方案，情况好转后再调整治疗。

（二）CR后的治疗

1. 治疗原则：①MRD持续阳性或上升的患者，CD19/CD3双特异性抗体（Blinatumomab，贝林妥欧单抗）清除残留后行异基因造血干细胞移植（allo-HSCT）。②MRD阴性或不详的患者，可继续多药联合化疗±CD19/CD3双特异性抗体巩固治疗，伴预后不良临床特征或遗传学异常的患者行allo-HSCT。

鼓励开展有科学依据的探索性前瞻性临床试验（如CAR-T细胞治疗或抗体药物偶联物清除MRD，巩固治疗采用化疗、免疫治疗、分子靶向治疗等多种治疗手段组合等）。

2. 治疗方案：缓解后强烈的巩固化疗和（或）抗体免疫治疗可清除残存的白血病细胞、提高疗效，但是巩固治疗方案在不同的研究组、不同的人群并不相同。一般应给予多疗程的治疗，药物组合包括诱导治疗使用的药物（如长春碱类药物、蒽环类药物、糖皮质激素等）、MTX、阿糖胞苷（Ara-C）、6-MP、ASP等。缓解后治疗可以包括1～2个疗程再诱导方案（如VDLP方案），MTX和Ara-C为基础的方案各2～4个疗程；条件允许的患者可包括多个疗程CD19/CD3双特异性抗体（可给予4～5个疗程，如果桥接HSCT，可给予1～2个疗程）。

儿童样化疗方案强调整个治疗过程参考儿童ALL方案的设计，强调非骨髓抑制性药物的应用（包括糖皮质激素、长春碱类、ASP）。

（1）一般应含有MTX方案：主要为大剂量MTX（HD-MTX）1～5 g/m^2^（T-ALL可以5 g/m^2^）。应用HD-MTX时应进行血清MTX浓度监测，甲酰四氢叶酸钙的解救治疗，至血清MTX浓度<0.1 µmol/L（或低于0.25 µmol/L，或根据本单位的界值决定）时结合临床症状停止解救（不能及时获取MTX浓度时，应关注血清肌酐的变化和黏膜损伤情况）。

（2）含有Ara-C为基础的方案。Ara-C可以为标准剂量、分段应用（如CTX、Ara-C、6-MP为基础的CAM方案），或中大剂量Ara-C为基础的方案。

（3）继续应用ASP，与其他药物（如MTX、Ara-C等）组成联合方案。

（4）缓解后6个月左右参考诱导治疗方案（VDLP）予再诱导强化一次。

（5）HSCT：考虑allo-HSCT的患者应在一定的巩固强化治疗后尽快移植。无合适供者的预后不良组患者（尤其是MRD持续阴性者）、预后良好组患者（MRD阴性者）可以考虑在充分的巩固强化治疗后进行自体造血干细胞移植（auto-HSCT），auto-HSCT后应继续予一定的维持治疗。

无移植条件的患者、持续属于预后良好组的患者可以按计划巩固强化治疗。

（6）老年患者可以适当调整治疗强度（如降低Ara-C、MTX、ASP等的用量）。

3. 注意事项：为减少复发、提高生存率，诱导治疗结束后应尽快开始缓解后的巩固强化治疗（诱导缓解治疗和缓解后治疗不要有过长的间歇期）。

应根据患者的危险度分组情况和MRD水平（详见MRD监测部分）判断是否需要行allo-HSCT，并需积极寻找供者。

（三）维持治疗

ALL患者强调维持治疗，基本方案：6-MP 60～75 mg/m^2^每日1次，MTX 15～20 mg/m^2^每周1次。

注意事项：①6-MP晚上用药效果较好。可以用硫鸟嘌呤（6-TG）替代6-MP。维持治疗期间应注意监测血常规和肝功能，调整用药剂量。②维持治疗既可以在完成巩固强化治疗之后单独连续使用,也可与强化巩固方案交替序贯进行。③自取得CR后总的治疗周期至少2年。

（四）特殊类型ALL的治疗

1. ETP-ALL的治疗：目前的经验证明采用ALL的传统诱导治疗方案（如VDCLP等）治疗ETP-ALL的CR率低、缓解质量差（MRD难以转阴）；单纯化疗的长生存率低。鼓励参加临床试验，取得CR后尽快行allo-HSCT[11]–[14]。

2. BCR::ABL1样ALL的治疗：BCR::ABL1样ALL的重要特点是存在涉及BCR::ABL1外的其他酪氨酸激酶易位（形成多种融合基因）、CRLF2易位和（或）JAK-STAT信号通路基因突变。

可以根据不同的分子学特点联合相应的靶向药物治疗，如涉及ABL系列融合基因的患者可以联合达沙替尼等酪氨酸激酶抑制剂（TKI）治疗。用药方法可以参考Ph^+^-ALL中TKI的使用方法[15]。

BCR::ABL1样ALL预后较差，应及早行allo-HSCT。

**Ph^−^-ALL推荐参考治疗方案**

1. 年轻成人和青少年（AYA）患者（<40岁）

（1）优先推荐方案：

•CALGB 10403方案[16]

•DFCI00-01为基础的DFCI ALL方案[17]

•IH-2014方案[18]

•PDT-ALL-2016方案[19]–[20]

（2）一般推荐方案：

•GRAALL-2005方案[21]

•Hyper-CVAD方案（MDACC）[22]–[23]

•ECOG1910方案（包含CD19/CD3双特异性抗体，CD20阳性使用利妥昔单抗）[24]

2. 成年患者（≥40岁，<60岁）

（1）优先推荐方案：

•ECOG1910方案（包含CD19/CD3双特异性抗体，CD20阳性使用利妥昔单抗）[24]

（2）一般推荐方案：

•IH-2014方案[18]

•CALGB8811方案[25]–[26]

•GRAALL-2005方案[21]

•Hyper-CVAD方案（MDACC）[22]–[23]

•MRC UKALLXII/ECOG E2993方案[27]

•Mini-HCVD方案+CD22抗体偶联药物（Inotuzumab ozogamicin, 奥加依托珠单抗）±CD19/CD3双特异性抗体[28]–[30]

3. 老年患者（≥60岁）

（1）低强度方案：

•长春碱类+泼尼松[31]

•长春碱类+泼尼松+6-MP+MTX（POMP方案）[32]

（2）中等强度方案：

•GRAALL方案[33]

•改良的DFCI老年ALL方案[34]

•Mini-HCVD方案+CD22抗体偶联药物±CD19/CD3双特异性抗体[28]–[30]

（3）高强度方案：

•Hyper-CVAD方案（Ara-C剂量减为1 g/m^2^）±CD19/CD3双特异性抗体[35]

•CALGB9111方案[36]

•ECOG 1910方案（包含CD19/CD3双特异性抗体，CD20阳性使用利妥昔单抗）[24]

二、Ph^+^-ALL的治疗

（一）成年患者（年龄<60岁）Ph^+^-ALL的治疗[29]–[34]

1. 诱导治疗：

（1）治疗原则：①临床试验。②多药化疗+ TKI治疗。③TKI+糖皮质激素±长春碱类。

（2）治疗方案：诱导化疗和Ph^−^-ALL一样，建议：①予VCR或长春地辛、蒽环/蒽醌类药物、糖皮质激素为基础的方案（如VDP）诱导治疗，可以联合CTX（组成VDCP方案）；②Hyper-CVAD方案；③鼓励进行临床研究。

一旦融合基因筛查（PCR方法）或染色体核型/FISH证实为Ph/BCR::ABL1阳性ALL（应明确转录本类型——P210、P190或少见类型转录本）则进入Ph^＋^-ALL治疗流程，可以不再应用ASP。自确诊之日起即加用（或根据方案设计尽早开始）TKI；优先推荐TKI持续应用的用药方式。对粒细胞缺乏（尤其是中性粒细胞绝对值<0.2×10^9^/L）持续时间较长（超过1周）、出现感染发热等严重并发症时，可以临时停用化疗和TKI，以减少患者严重感染风险。

（3）注意事项：诱导治疗第14天复查骨髓，根据骨髓（造血恢复和原始细胞比例）和血常规调整第3周的治疗。诱导治疗第28（＋7）天评估疗效，复查骨髓形态学、细胞遗传学（诊断时有异常者）、BCR::ABL1融合基因定量及流式MRD。有HSCT条件者，行HLA配型、积极寻找供者。

诱导治疗也可以在保证TKI用药的前提下适当降低化疗强度（如采用长春碱类药物、糖皮质激素联合TKI的方案），以保证患者安全。尽早开始腰穿、鞘注。

2. CR后的治疗：Ph^+^-ALL的缓解后治疗原则上参考一般Ph^−^-ALL的治疗（但可以不再使用ASP），应保证TKI的用药（TKI优先推荐持续应用，至维持治疗结束）；无条件应用TKI或多种TKI不耐受的患者按一般Ph^−^-ALL的方案治疗。成年患者（年龄<60岁）Ph^+^-ALL的缓解后化疗强度应有一定的保证（基本同Ph^−^-ALL）。

（1）有合适供者的患者建议选择allo-HSCT，合并其他不良预后因素者优先选择allo-HSCT（如出现ABL1激酶突变、流式细胞术MRD持续阳性或融合基因定量持续达不到主要分子学缓解、MRD指标呈上升趋势）。移植后继续用TKI维持治疗（使用时间为1～2年）。

（2）BCR::ABL1融合基因转阴性者（尤其是3个月内转阴性者），可以考虑auto-HSCT，移植后予TKI维持治疗。

（3）MRD阳性的Ph^+^-ALL患者可以采用CD19/CD3双特异性抗体±TKI清除残留病细胞后行allo-HSCT[37]，也可以进行探索性研究。MRD阴性患者也可以在巩固治疗中采用TKI+CD19/CD3双特异性抗体[38]。无合适供者的患者，按计划继续CD19/CD3双特异性抗体+TKI和(或)多药化疗+TKI治疗。条件允许的患者可给予4～5个疗程CD19/CD3双特异性抗体，如果桥接HSCT，可给予1～2个疗程。

（4）治疗过程中应定期监测BCR::ABL1融合基因水平（推荐定量检测）和流式MRD，MRD出现波动者应及时进行allo-HSCT。

（5）CNSL的预防治疗参考Ph^−^-ALL患者。

3. 维持治疗：

（1）可以应用TKI治疗者，采用TKI为基础的维持治疗（可以联合VCR、糖皮质激素，或6-MP和MTX；或干扰素），至CR后至少2年，其后可用TKI维持治疗。

（2）不能坚持TKI治疗者，采用干扰素（可以联合VCR、糖皮质激素）维持治疗，300万U/次，隔日1次，缓解后至少治疗2年。或参考Ph^−^-ALL进行维持治疗。

（二）老年Ph^+^-ALL（年龄≥60岁）的治疗

老年Ph^+^-ALL的治疗原则以TKI为基础，化疗参考老年Ph^−^-ALL。TKI优先推荐持续应用，至维持治疗结束[35]–[36]。

1. 诱导治疗：①临床试验。②TKI+多药化疗，如VDP，miniHCVD等方案。③TKI+糖皮质激素±长春碱类

2. CR后的治疗：继续TKI+糖皮质激素，或TKI+化疗巩固（可以参考上述方案的缓解后治疗），MRD阳性的患者可以采用CD19/CD3双特异性抗体±TKI清除残留病细胞[37]，也可以进行探索性研究。MRD阴性患者也可以在巩固治疗中采用TKI+CD19/CD3双特异性抗体[38]。有移植意愿、合适供者的患者（尤其是伴有其他预后不良因素者）可以选择allo-HSCT。条件允许的患者可给予4～5个疗程CD19/CD3双特异性抗体，如果桥接HSCT，可给予1～2个疗程。

3. 维持治疗：基本和年轻患者相同，采用TKI为基础的维持治疗。

三、CNSL的诊断、预防和治疗

CNSL是急性白血病（尤其是ALL）复发的主要根源之一，严重影响ALL的疗效。诊断时有神经系统症状者应先进行头颅影像学检查（CT或MRI检查），排除出血或占位后再考虑腰穿，无神经系统症状者按计划进行CNSL的预防。有条件的医疗机构应尽可能采用流式细胞术进行脑脊液检测[39]–[41]。

（一）CNSL状态分类

CNS-1：白细胞分类无原始淋巴细胞（不考虑脑脊液白细胞计数）。

CNS-2：脑脊液白细胞计数<5个/µl，可见原始淋巴细胞。

CNS-3：脑脊液白细胞计数≥5个/µl，可见原始淋巴细胞。

（二）CNSL诊断标准

目前CNSL尚无统一诊断标准。1985年讨论关于ALL预后差的危险因素时，提出CNSL下列诊断标准：脑脊液白细胞计数≥0.005×10^9^/L（5个/µl），离心标本证明细胞为原始细胞者，即可诊断CNSL。

流式细胞术检测脑脊液在CNSL中的诊断意义尚无一致意见，但出现阳性应按CNSL对待[42]。

（三）CNSL的预防

任何类型的成人ALL均应强调CNSL的早期预防。预防措施包括：①鞘内化疗；②放射治疗；③大剂量全身化疗；④多种措施联合应用。

1. 鞘内化疗：鞘内化疗是预防CNSL的主要措施。诱导治疗过程中没有中枢神经系统症状者可以在血细胞计数安全水平后行腰穿、鞘注。鞘内注射主要用药包括：地塞米松、MTX、Ara-C。常用剂量为MTX 10～15 mg/次、Ara-C 30～50 mg/次、地塞米松5～10 mg/次三联（或两联）用药。

巩固强化治疗中也应进行积极的CNSL预防，主要是腰穿、鞘注（鞘注总次数一般应达12次以上，拟行HSCT的患者一般在HSCT前应完成4～6次），鞘注频率一般不超过2次/周。

2. 预防性颅脑放疗：目前已较少采用预防性颅脑放疗。18岁以上的高危组患者或40岁以上（不考虑HSCT）的患者可考虑预防性颅脑放疗，放疗一般在缓解后的巩固化疗期或维持治疗时进行。预防性照射部位一般为单纯颅脑，总剂量1 800～2 000 cGy，分次完成。

（四）CNSL的治疗

确诊CNSL的ALL患者，尤其是症状和体征明显者，建议先行腰穿、鞘注，每周2次，直至脑脊液正常；以后每周1次×4～6周。

也可以在鞘注化疗药物至脑脊液白细胞计数正常、症状体征好转后再行放疗（头颅+脊髓放疗）。建议头颅放疗剂量2 000～2 400 cGy、脊髓放疗剂量1800～2 000 cGy，分次完成。进行过预防性头颅放疗的患者原则上不进行二次放疗。

四、难治复发ALL的治疗

（一）难治复发Ph^−^-ALL

难治复发Ph^−^-ALL的治疗目前无统一意见，可以选择的方案如下。

1. 临床试验：如新药临床试验，各种靶点的CAR-T细胞治疗（如靶向CD19、CD22、CD20的单靶点或双靶点CAR-T细胞治疗B-ALL，靶向CD7、CD5的CAR-T细胞治疗T-ALL等）及研究者发起的临床研究（CD38单抗治疗CD38阳性的ALL、西达本胺为基础的T-ALL方案，BCL-2抑制剂的应用等）[44]–[49]。

2. 难治复发B-ALL可以根据流式抗原表达考虑CD19 CAR-T细胞（Inaticabtagene Autoleucel，纳基奥仑赛）[43]、CD22抗体偶联药物[44]或CD19/CD3双特异性抗体[45]为基础的挽救治疗。

3. 如不能参加临床试验或接受免疫治疗，可进行多药联合化疗（具体见推荐方案）。

（二）难治复发Ph^+^-ALL

1. 临床试验：如新药临床试验，各种靶点的CAR-T细胞治疗（如靶向CD19、CD22、CD20的单靶点或双靶点CAR-T细胞等）及研究者发起的临床研究（如BCL-2抑制剂为基础的方案等）等。

2. 规范应用TKI为基础的治疗中复发、难治的患者：以ABL1激酶区突变结果、前期用药情况为依据，选择适合的TKI药物。可以继续联合化疗（参考初诊患者的诱导治疗方案）。

3. 可以根据流式抗原表达及ABL1激酶区突变结果考虑CD19/CD3双特异性抗体±TKI、CD22抗体偶联药物±TKI、CD19 CAR-T细胞治疗为基础的挽救治疗。

4. 如不能参加临床试验或接受免疫治疗，且无敏感TKI选择的患者可以采用复发难治Ph^−^-ALL的治疗方案。

无论是Ph^−^-ALL、还是Ph^+^-ALL，在挽救治疗的同时即应考虑HSCT，及时寻找供者，达到再次缓解后进行allo-HSCT。

**推荐化疗方案：**

1. MopAD方案[46]

2. Augmented Hyper-CVAD[47]

3. 氟达拉滨为基础的方案：FLAG-IDA[48]、FLAM[49]

4. 复发难治T-ALL可考虑奈拉滨联合依托泊苷、CTX[50]

## 第三部分 ALL治疗反应定义、监测和随访

一、ALL治疗反应的定义

（一）骨髓和外周血疗效标准

1. CR：①外周血无原始细胞，无髓外白血病；②骨髓三系造血恢复，原始细胞<5％；③中性粒细胞绝对计数（ANC）≥1.0×10^9^/L；④PLT≥100×10^9^/L。

2. CR伴部分血液学恢复（CRh）：PLT≥50×10^9^/L且ANC≥0.5×10^9^/L。其他应满足CR的标准。

3. CR伴不完全血液学恢复（CRi）：PLT<100×10^9^/L且ANC≥1.0×10^9^/L，或者PLT≥100×10^9^/L且ANC<1.0×10^9^/L。其他应满足CR的标准。

4. 形态学无白血病状态（MLFS）：①原始细胞<5％，且无髓外白血病；②PLT<50×10^9^/L且ANC<0.5×10^9^/L；③骨髓细胞成分≥10％，骨髓标本取材合格且至少计数200个细胞。

5. 再生障碍性骨髓：骨髓细胞成分<10％和（或）无法计数200个细胞，其他符合MLFS标准。

6. 难治性疾病：诱导治疗结束（一般指4周方案或Hyper-CVAD方案）未能取得CR/CRi/CRh。

7. 疾病进展（PD）：外周血出现白血病细胞，或外周血或骨髓原始细胞绝对数增加25％以上，或出现髓外疾病。

8. 疾病复发：已取得CR的患者外周血或骨髓又出现原始细胞（比例≥5％），或出现髓外疾病。

（二）CNSL的治疗反应

1. CNS缓解：CNS-2或CNS-3患者达到CNS-1状态。

2. CNS复发：新出现的CNS-2或CNS-3状态或出现CNSL的临床症状（如面神经麻痹、脑/眼受累，或下丘脑综合征的表现）。

（三）髓外疾病的治疗反应

髓外疾病的疗效判断依靠颈部/胸部/腹部/盆腔CT和（或）PET-CT。

CR：CT检查淋巴瘤性肿块完全消失；或肿块大小仍有残留但PET阴性。

部分缓解（PR）：淋巴瘤性肿块最大垂直直径（SPD）乘积之和缩小>50％，治疗前PET阳性的患者，治疗后至少一个前期累及部位PET阳性。

疾病进展（PD）：淋巴瘤性肿块SPD增加>25％，治疗前PET阳性的患者，治疗后至少一个前期累及部位PET阳性。

未缓解（NR）：不满足PR或PD标准。

复发：取得CR的患者又出现淋巴瘤性肿块。

二、MRD的监测和完成治疗后的随访

1. ALL整个治疗期间应强调规范的MRD监测，并根据监测结果进行动态的危险度分层和治疗方案调整[51]–[52]。

（1）早期：诱导治疗期间（第14天）和（或）结束时（第28天左右）。

（2）缓解后定期监测：应至少保证治疗3个月（第12～16周）、6个月（第24～28周）及巩固治疗结束时的MRD监测。

MRD监测可用于预后评估和危险度、治疗策略的调整；缓解后MRD水平持续较高或治疗过程中MRD由阴性转为阳性的患者具有较高的复发危险（危险度上调），缓解后治疗应进行调整（如allo-HSCT）。

2. MRD的监测方法：

（1）经典的MRD检测技术：①IgH、TCR定量PCR检测（DNA水平）；②4～6色流式细胞术MRD检测；③融合基因转录本的实时定量PCR（如BCR::ABL1）。

（2）新的高通量MRD检测技术：①基于EuroFlow≥8色二代流式细胞术检测MRD；②IgH、TCR高通量测序。

3. Ph^+^-ALL疾病反复时应注意进行ABL1激酶区突变的分析。

4. 完成巩固强化治疗后的随访检查：

（1）第1年每1～2个月1次，检查项目包括体格检查、血常规、肝功能（尤其是服用6-MP的患者）。

（2）第2年每3～6个月1次，检查项目同第1年。

（3）第3年及以后每6～12个月1次或根据病情需要。如持续缓解状态一般至诊断后5年可以停止复查。

每个复查随访的时间点均应包括骨髓形态学和MRD（流式细胞术MRD和/或特异融合基因定量）的检测。
